# Patients undergoing double J substitution with a pigtail suture stent report a significant decrease of stent-related symptoms. Results from a prospective multicenter longitudinal trial

**DOI:** 10.1007/s00345-024-04879-5

**Published:** 2024-03-22

**Authors:** Andrea Bosio, Stefania Ferretti, Eugenio Alessandria, Federico Vitiello, Eugenia Vercelli, Davide Campobasso, Luca Micai, Claudia Gozzo, Glauco Bertello, Giulio Gaetano Guarino, Claudia Alice, Alessandro Bisconti, Fabrizio Fop, Paolo Gontero

**Affiliations:** 1 Department of Urology, AOU Città della Salute e della Scienza, Molinette University Hospital, Turin, Italy; 2 Department of Surgical Sciences, University of Turin, Turin, Italy; 3 Department of Urology, AOU University Hospital, Parma, Italy; 4 Department of Urology, AOU University Hospital, Modena, Italy; 5 Department of Nephrology, AOU Città della Salute e della Scienza, Molinette University Hospital, Turin, Italy

**Keywords:** Double J, Pigtail suture stents, Stent-related symptoms, Ureteroscopy, URS, LUTS, Pain, RIRS, Stents, Urinary calculi, Urolithiasis, Ureteral stent, Stent-related discomfort, EndoUrology, Urology

## Abstract

**Purpose:**

To compare stent-related symptoms (SRS) in patients with double J (DJ) undergoing substitution with a pigtail suture stent (PSS) after ureteroscopy (URS), through the Ureteral Stent Symptom Questionnaire (USSQ).

**Materials and methods:**

Patients with DJ undergoing URS for stone treatment were enrolled in this prospective multicenter longitudinal study. The USSQ was submitted thrice: 2 weeks after DJ, 2 weeks after PSS and 4 weeks after PSS removal (baseline). Primary endpoint: to compare Urinary Symptom Index Score and the rate of patients with pain 2 weeks after DJ and PSS. Secondary endpoints: to compare other USSQ scores and single answers 2 weeks after DJ and PSS, and DJ and PSS USSQ scores with baseline.

**Results:**

93 patients were enrolled. 2 weeks Urinary Symptom Index Score (*p* < 0.001) and the percentage of patients complaining of pain (60.2% vs 88.2%, *p* < 0.001) were significantly in favour of PSS compared to DJ. 2 weeks scores were significantly improved with PSS compared to DJ: Pain Index (*p* < 0.001), VAS (*p* < 0.001), General Health Index (*p* < 0.001) and Work Performance Index (*p* < 0.001). All urinary symptoms were significantly decreased with PSS, including renal pain during micturition and pain interfering with life. Pain Index Score (*p* = 0.622) and VAS (*p* = 0.169) were comparable to baseline with PSS, while differed with DJ.

**Conclusions:**

Patients undergoing DJ substitution with PSS after URS report a significant decrease of SRS. Urologists may consider positioning PSS after URS in pre-stented patients to reduce the impact of SRS.

## Introduction

Double J (DJ) ureteral stents are commonly used in urological practice [1]. However, the significant impact of stent-related symptoms (SRS) on urinary symptoms, pain and quality of life has been widely documented [2, 3]. Countless solutions have been proposed to provide more tolerable stents, both investigating pharmacological [4] and design aspects [5]. Vogt and colleagues introduced pigtail suture stents (PSS) in 2015, replacing the DJ distal pigtail with a suture thread reaching the bladder [6]. This device was later investigated by randomized controlled studies (RCT) which assessed more tolerability compared to conventional DJ [7–10].

In their initial experience, Vogt and colleagues compared SRS in 24 patients complaining of DJ-related symptoms, undergoing substitution with a self-made PSS: a significant decrease of SRS was shown [6]. Neither further studies investigated this scenario, nor commercially available PSS have ever been involved in this setting. Hence, we performed a prospective multicenter longitudinal study to evaluate patients undergoing DJ substitution with a commercially available PSS after ureteroscopy (URS) for stone treatment, making a comparison of symptoms related to the two different devices.

## Materials and methods

### Trial design and participants

Patients with indwelling DJ undergoing URS for stone treatment, were asked to participate in a prospective longitudinal multicenter study. Reasons for primary DJ placement were renal colic with concomitant urinary tract infection and unsuccessful attempt of primary URS (e.g., due to narrow ureter). Inclusion criteria were: patients with indwelling DJ undergoing URS for renal or ureteral stones treatment; WHO performance status 0–2; patients aged 18–80. Exclusion criteria were: intraprocedural complications (e.g., ureteral damage) during URS, urinary tract anatomical abnormalities, solitary kidney, chronic hydronephrosis with impaired function of the kidney.

Patients were enrolled and underwent surgery at University Hospitals of Turin (TO)—Città della Salute e della Scienza—and Parma (PR), Italy. The trial was conducted according to the Declaration of Helsinki [11]. All the involved patients gave their written consent. According to Italian law, Institutional Review Board approval was waived due to the observational nature of the research.

### Intervention

Patients with indwelling conventional hydrophilic DJ stent (Vortek®, Coloplast—TO, Percuflex™, Boston Scientific—PR) underwent URS not before 2 weeks after DJ placement. URS was performed with a 7 Fr semirigid ureteroscope (Karl Storz, Tuttlingen, D) and flexible URS with a Flex-X2 (Karl Storz, Tuttlingen, D) fiberoptic flexible ureteroscope, with or without ureteral access sheath placement. Lithotrispy was performed using Ho:YAG laser (30W Rocamed, Monaco, MC in TO—35W Quanta System, Samarate, IT in PR) and extraction of residual fragments using a 0-tip nitinol basket.

**Fig. 1:**
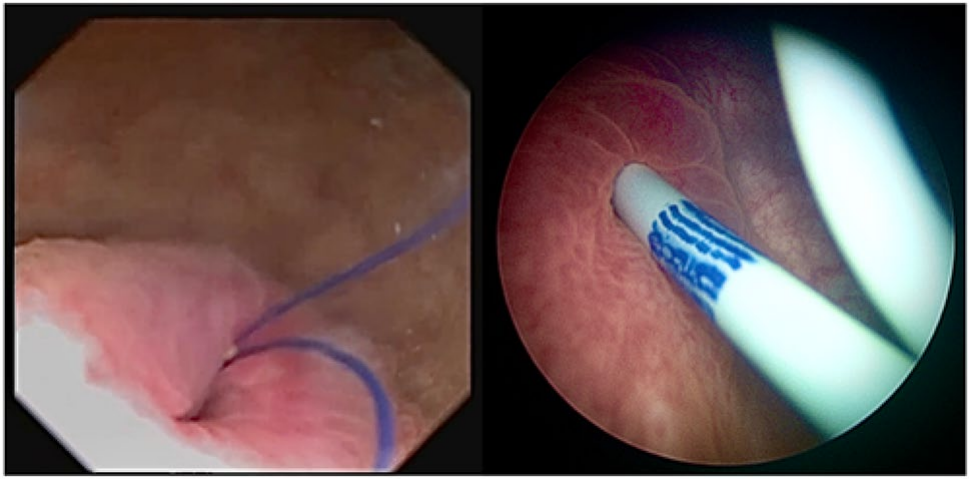
PSS threads and DJ distal end: cystoscopy view

After the procedure, a commercially available PSS (JFil®—Rocamed, Monaco, MC) was inserted in all cases, featuring a 7 Fr × 16 cm single renal pigtail stent, with the distal part ending in a fluted beak connected to a 0.3 Fr double surgical thread reaching the bladder (Fig. 1). PSS was removed through flexible cystoscope 2 weeks after surgery.

### Follow-up

SRS were assessed through the validated Italian version [12] of the Ureteral Stent Symptoms Questionnaire (USSQ) [2]. The USSQ was submitted thrice: 2 weeks after DJ placement (before URS), 2 weeks after PSS placement (before PSS removal) and 4 weeks after PSS removal (considered as baseline).

### Endpoints

Primary endpoint was to compare Urinary Symptom Index Score (sum of USSQ Urinary symptoms section) and the rate of patients complaining of pain (question P1 of USSQ) 2 weeks after DJ and PSS placement.

Secondary endpoints were to compare other USSQ scores and USSQ single answers 2 weeks after DJ and PSS placement, and DJ and PSS USSQ domains’ scores and single answers with baseline.

### Statistical analysis

Continuous variables were presented as median (interquartile ranges—IQR), while categorical variables as frequency. The association between categorical variables was analyzed through Pearson’s Chi-Squared and McNemar’s tests, while Wilcoxon matched-pairs signed-ranks test was used for continuous variables. All the reported *p*-values were obtained by the two-sided exact method at the conventional 5% significance level. No information was available for the longitudinal variability of paired data in the design phase. We hypothesized a 3-point difference in the primary endpoint (Urinary Symptom Index Score) between the two groups (DJ versus PSS). By setting the power at 80% and *α* risk at 0.05, the minimum number of patients resulted 73. 93 patients were enrolled in the study period, with Urinary Symptom Index Score variation of 10 points, obtaining a final power approximating 100%. All statistical analyses were performed using Spss (IBM Corp. Released 2021. IBM SPSS Statistics for Windows, Version 28.0. Armonk, NY: IBM Corp.).

## Results

93 patients with indwelling DJ underwent PSS insertion after URS between April 2020 and March 2023, and were included in this prospective multicenter longitudinal study. 70 patients were treated in TO and 23 in PR. All 93 patients completed and returned their USSQ.

### Patients and stones

Patients’ median age was 61 years [48, 68], median height 174 cm [165, 178], median weight 78 kg [70, 85] and median BMI 26 kg/mq [23, 29]. Most patients were male (72%; 67/93). Median stone size was 9 mm [6, 11] and most stones were located in the kidney (58.2%—53/91). The characteristics of patients and stones are shown in Table 1.

**Table 1:** Characteristics of patients and stones

Patients	Median [IQR]
Age (years)	61 [48, 68]
Height (cm)	174 [165, 178]
Weight (kg)	78 [70, 85]
BMI (kg/m^2^)	25.95 [23, 29]

	Number (%)
Gender	
Male	67/93 (72%)
Female	26/93 (28%)

Stones	Median [IQR]
Size (mm)	9 [6, 11]

	Number (%)
Location	
Kidney	53/91 (58.2%)
UPJ	12/91 (13.2%)
Lumbar ureter	17/91 (18.7%)
Iliac ureter	6/91 (6.6%)
Pelvic ureter	3/91 (3.3%)

### Stent-related symptoms: DJ versus PSS

Both 2 weeks Urinary Symptom Index Score (*p* < 0.001) and the percentage of patients complaining of body pain and discomfort (60.2% vs 88.2%, *p* < 0.001) showed significant difference in favor of PSS compared to DJ. 2 weeks USSQ domains’ scores were also significantly improved with PSS, in particular Pain Index Score (*p* < 0.001), VAS Score (*p* < 0.001), General Health Index Score (*p* < 0.001) and Work Performance Index Score (*p* < 0.001). No differences were noticed concerning Sexual Matters Score (*p* = 0.059). While having PSS, patients also reported a better feeling in case of a further stenting in the future (USSQ question GQ, *p* < 0.001). USSQ domains’ scores at 2 weeks with DJ and PSS are reported in Table 2.

**Table 2:** USSQ domains’ scores at 2 weeks with DJ and PSS

USSQ scores for each section at 2 weeks	DJ *N* = 93	PSS *N* = 93	
	Median [IQR]	Median [IQR]	*p*^a^
Urinary Symptom Index Score (U1–U11)	32 [27, 37]	22 [19, 28]	**< 0.001**
Pain Index Score (P3–P9)^b^	21 [17, 25]	16 [12, 20]	**< 0.001**
Pain Intensity—VAS Score (P3)^b^	6 [4, 8]	4 [2, 6]	**< 0.001**
General Health Index Score (G1–G6)	15 [10, 18]	11 [9, 15]	**< 0.001**
Work Performance Index Score (W5–W7)	7 [4, 10]	4 [3, 7]	**< 0.001**
Sexual Matters Score (S3–S4)	4 [3, 5]	3 [3, 4]	0.059
Feeling about stenting in the future (GQ)^c^	6 [4, 7]	4 [3, 6]	**< 0.001**

	Number (%)	Number (%)	*p*^d^
Body pain or discomfort (P1)	82/93 (88.2)	56/93 (60.2)	**< 0.001**
No active sex life with the stent in situ (S1)^e^	39/93 (41.9)	40/91 (44.0)	0.617
Sex life stopped because of the stent (S2)^e^	16/58 (27.6)	11/52 (21.2)	0.655

^a^“*p*” values were obtained using the Wilcoxon test

^b^Pain Index and VAS (Visual Analogue Scale) scores refer only to patients with body pain (who answered “yes” to question P1)

^c^Question GQ: “in the future, if you were advised to have another stent inserted, how would you feel about it?” Answers: 4 “mixed feelings”, 5 “mostly dissatisfied”

^d^“*p*” values were obtained using McNemar’s Chi-Squared test

^e^A few patients did not answer to questions about sexual matters

Statistical significant *p* values are reported in bold

Considering single answers, all urinary symptoms resulted to be significantly decreased with PSS: urinary frequency (*p* < 0.001), nocturia (*p* < 0.001), urgency (*p* < 0.001), urge incontinence (*p* < 0.001), incontinence without urge (*p* = 0.021), feeling of incomplete bladder emptying (*p* < 0.001), burning at voiding (*p* < 0.001), macroscopic hematuria (*p* < 0.001), grade of hematuria (*p* < 0.001). Urinary symptoms were less a problem (*p* < 0.001) with PSS and feeling about the future was better (*p* < 0.001). Renal pain while passing urine (*p* = 0.001) and pain interfering with life (*p* = 0.001) were also reduced with PSS. Patients with PSS experienced less difficulty in performing both light (*p* < 0.001) and heavy (*p* = 0.001) physical activities, felt tired or worn out less frequently (*p* < 0.001), enjoyed more their social life (*p* < 0.001) and needed less extra help from their family members or friends (*p* < 0.001). The number of days spent in bed due to SRS (*p* < 0.001) were also reduced with PSS, together with the need for cutting down routine activities (*p* = 0.001), frequent rests at work (*p* = 0.001), changes in usual job (*p* = 0.001) and decreasing the regular number of hours at work (*p* = 0.006). Pain during sexual intercourse (*p* = 0.028), symptoms of urinary tract infection (*p* < 0.001), need for antibiotics (*p* = 0.001) and the need to seek help of a health professional due to any problem associated with the stent (*p* < 0.001) and need to visit the hospital (*p* = 0.003) were also significantly reduced with PSS compared to DJ. Results of 2 weeks USSQ single answers with DJ and PSS are reported in Table 3.

**Table 3:** USSQ single answers at 2 weeks with DJ and PSS

	DJ	PSS	
	Median [IQR]	Median [IQR]	*p*^a^
**Urinary symptoms**			
Urinary frequency (U1)	4 [3, 5]	3 [2, 3]	**< 0.001**
Nocturia (U2)	3 [3, 4]	2 [2, 3]	**< 0.001**
Urgency (U3)	3 [2, 4]	2 [1, 3]	**< 0.001**
Urge incontinence (U4)	2 [1, 3]	1 [1, 2]	**< 0.001**
Incontinence without urge (U5)	1 [1, 1]	1 [1, 1]	**0.021**
Incomplete emptying (U6)	3 [2, 4]	2 [1, 2]	**< 0.001**
Burning at voiding (U7)	3 [2, 4]	1 [1, 3]	**< 0.001**
Macroscopic hematuria (U8)	2 [1, 3]	1 [1, 2]	**< 0.001**
Grade of hematuria (U9)	2 [1, 3]	1 [1, 2]	**< 0.001**
Current state is a problem (U10)	4 [2, 4]	2 [1, 3]	**< 0.001**
Rest of life like this (U11)^b^	6 [5, 7]	5 [3, 6]	**< 0.001**
**Body pain or discomfort**^c^			
Pain during physical activities (P4)^c^	3 [3, 4]	3 [2, 3]	**0.001**
Pain causing sleep interruption (P5)^c^	2 [1, 3]	1 [1, 2.75]	**0.036**
Pain while passing urine (P6)^c^	3 [2, 4]	2 [1, 3]	**< 0.001**
Renal pain while passing urine (P7)^c^	2 [1, 2]	1 [1, 2]	**< 0.001**
Pain requiring painkillers (P8)^c^	2 [1, 3]	2 [1, 2]	**0.009**
Pain interfering with life (P9)^c^	4 [2, 4]	3 [2, 3]	**0.001**
**General health**			
Difficulty in light physical activities (G1)	2 [1, 3]	1 [1, 2]	**< 0.001**
Difficulty in heavy physical activities (G2)	2 [1, 4]	2 [1, 3]	**0.001**
Felt tired or worn out (G3)	3 [2, 4]	2 [2, 3]	**< 0.001**
Felt calm and peaceful (G4)	3 [2, 4]	2 [1, 3]	**0.020**
Enjoyed social life (G5)	3 [2, 4]	2 [1, 3]	**< 0.001**
Needed extra help from family or friends (G6)	2 [1, 3]	1 [1, 2]	**< 0.001**
**Work performance**			
Days in bed due to symptoms (W2)	2 [0, 5.5]	0 [0, 1]	**< 0.001**
Decrease in routine activities (W3)	3 [0, 13.25]	0 [0, 3]	**< 0.001**
Frequent rests at work (W5)	2 [1, 3]	2 [1, 2]	**0.001**
Changes in usual job (W6)	2 [1, 3]	1 [1, 2]	**0.001**
Decrease in hours of work (W7)	2 [1, 3.75]	1 [1, 2]	**0.006**
**Sexual matters**			
Pain during sexual intercourse (S3)	2 [1, 2.25]	1 [1, 1]	**0.028**
Satisfaction with sex life (S4)	2 [2, 3]	2 [2, 2]	0.430
**Additional problems**			
Feeling of urinary tract infection (A1)	1 [1, 3]	1 [1, 2]	**< 0.001**
Need for antibiotics (A2)	1 [1, 2]	1 [1, 1]	**0.001**
Need for health professional help (A3)	1 [1, 2]	1 [1, 1]	**< 0.001**
Need to visit the hospital (A4)	1 [1, 1]	1 [1, 1]	**0.003**

^a^“*p*” values were obtained using the Wilcoxon test

^b^Question U11: “if you were to spend the rest of your life with the urinary symptoms, if any, associated with the stent just the way they are, how would you feel about it?” Answers: 5 “mostly dissatisfied”, 6 “unhappy”

^c^Pain scores rates refer only to the patients with pain

Statistical significant *p* values are reported in bold

### Stent-related symptoms: DJ and PSS compared to baseline

Two weeks USSQ domains’ scores significantly differed from baseline both with DJ and PSS, regarding Urinary Symptom Index Score (*p* < 0.001), body pain and discomfort (P1 of the USSQ) (*p* < 0.001 and *p* < 0.001), General Health Index Score (*p* < 0.001 and *p* = 0.001, respectively) and Work Performance Index Score (*p* < 0.001 and *p* = 0.036). Pain Index Score (*p* = 0.622), VAS Score (*p* = 0.169), Sexual Matters Score (*p* = 0.565) and feeling about possible stenting in future (GQ, *p* = 0.204) with PSS were comparable to baseline, while significantly differed from baseline with DJ (*p* = 0.009, *p* = 0.014, *p* = 0.011, *p* < 0.001, respectively). USSQ domains’ scores at 2 weeks with DJ and PSS compared to baseline are reported in Table 4.

**Table 4:** USSQ domains’ scores at 2 weeks with DJ and PSS compared with baseline

	DJ		PSS		Baseline
	Median [IQR]	*p*^a^	Median [IQR]	*p*^b^	Median [IQR]
Urinary Symptom Index Score (U1–U11)	32 [27, 37]	**< 0.001**	22 [19, 28]	**< 0.001**	17 [14, 23]
Pain Index Score (P3–P9)^c^	21 [17, 25]	**0.009**	16 [12, 20]	0.622	15 [11, 21]
Pain Intensity—VAS Score (P3)^c^	6 [4, 8]	**0.014**	4 [2, 6]	0.169	4 [3, 6.5]
General Health Index Score (G1–G6)	15 [10, 18]	**< 0.001**	11 [9, 15]	**0.001**	9 [7, 13]
Work Performance Index Score (W5–W7)	7 [4, 10]	**< 0.001**	4 [3, 7]	**0.036**	3 [3, 6.75]
Sexual Matters Score (S3–S4)	4 [3, 5]	0.302	3 [3, 4]	0.783	3 [2, 4]
Feeling about stenting in the future (GQ)^d^	6 [4, 7]	**< 0.001**	4 [3, 6]	0.204	5 [4, 6]

	Number (%)	*p*^e^	Number (%)	*p*^f^	Number (%)
Body pain or discomfort (P1)	82/93 (88.2)	**< 0.001**	56/93 (60.2)	**< 0.001**	27/79 (34.2)

^a^“*p*” values refer to symptoms at 2 weeks with DJ compared with baseline and were obtained using the Wilcoxon test

^b^“*p*” values refer to symptoms at 2 weeks with PSS compared with baseline and were obtained using the Wilcoxon test

^c^Pain Index and VAS (Visual Analogue Scale) Scores refer only to patients with body pain (who answered “yes” to question P1)

^d^Question GQ: “in the future, if you were advised to have another stent inserted, how would you feel about it?” Answers: 4 “mixed feelings”, 5 “mostly dissatisfied”

^e^“*p*” values refer to symptoms at 2 weeks with DJ compared with baseline and were obtained using McNemar’s Chi-Squared test

^f^“*p*” values refer to symptoms at 2 weeks with PSS compared with baseline and were obtained using McNemar’s Chi-Squared test

^g^A few patients did not answer to questions about sexual matters

Statistical significant *p* values are reported in bold

All single urinary symptoms with both DJ and PSS showed significant differences compared to baseline, except for incontinence without urge and incomplete emptying, which were comparable with PSS (*p* = 0.166 and *p* = 0.167). The following symptoms were significantly worsened with DJ, but not with PSS, compared to baseline: pain during physical activities (P4), pain while passing urine (P6), renal pain while passing urine (P7), pain interfering with life (P9), difficulty in light and heavy physical activities (G1–G2), feeling tired or worn out (G3), enjoying social life (G5), need for extra help from family members or friends (G6), days in bed due to SRS (W2), decrease in routine activities (W3), frequent rests at work (W5), changes in usual work (W6), pain during sexual intercourse (S3), symptoms of urinary tract infection (A1). Comparison of USSQ single answers with DJ and PSS versus baseline are reported in Table 5.

**Table 5:** USSQ single answers at 2 weeks with DJ and PSS compared with baseline

	DJ		PSS		Baseline
	Median [IQR]	*p*^a^	Median [IQR]	*p*^b^	Median [IQR]
**Urinary symptoms**					
Urinary frequency (U1)	4 [3, 5]	**< 0.001**	3 [2, 3]	**0.002**	2 [1, 3]
Nocturia (U2)	3 [3, 4]	**< 0.001**	2 [2, 3]	**< 0.001**	2 [1, 3]
Urgency (U3)	3 [2, 4]	**< 0.001**	2 [1, 3]	**0.001**	2 [1, 2]
Urge incontinence (U4)	2 [1, 3]	**< 0.001**	1 [1, 2]	**0.010**	1 [1, 1.5]
Incontinence without urge (U5)	1 [1, 1]	**0.004**	1 [1, 1]	0.166	1 [1, 1]
Incomplete emptying (U6)	3 [2, 4]	**< 0.001**	2 [1, 2]	0.167	1 [1, 2]
Burning at voiding (U7)	3 [2, 4]	**< 0.001**	1 [1, 3]	**0.044**	1 [1, 2]
Macroscopic hematuria (U8)	2 [1, 3]	**< 0.001**	1 [1, 2]	**0.021**	1 [1, 1]
Grade of hematuria (U9)	2 [1, 3]	**< 0.001**	1 [1, 2]	**0.009**	1 [1, 1]
Current state is a problem (U10)	4 [2, 4]	**< 0.001**	2 [1, 3]	**< 0.001**	1 [1, 2]
Rest of life like this (U11)^c^	6 [5, 7]	**< 0.001**	5 [3, 6]	**< 0.001**	3 [1, 5]
**Body pain or discomfort**^d^					
Pain during physical activities (P4)^d^	3 [3, 4]	**0.050**	3 [2, 3]	0.521	3 [1.75, 4]
Pain causing sleep interruption (P5)^d^	2 [1, 3]	0.719	1 [1, 2.75]	0.276	1 [1, 3]
Pain while passing urine (P6)^d^	3 [2, 4]	**0.011**	2 [1, 3]	0.918	2 [1, 3]
Renal pain while passing urine (P7)^d^	2 [1, 2]	**0.035**	1 [1, 2]	0.317	1 [1, 2]
Pain requiring painkillers (P8)^d^	2 [1, 3]	0.088	2 [1, 2]	0.527	1 [1, 2]
Pain interfering with life (P9)^d^	4 [2, 4]	**0.001**	3 [2, 3]	0.221	2 [1, 3]
**General health**					
Difficulty in light physical activities (G1)	2 [1, 3]	**< 0.001**	1 [1, 2]	**0.044**	1 [1, 2]
Difficulty in heavy physical activities (G2)	2 [1, 4]	**< 0.001**	2 [1, 3]	**0.011**	1 [1, 2]
Felt tired or worn out (G3)	3 [2, 4]	**< 0.001**	2 [2, 3]	0.107	2 [1, 2]
Felt calm and peaceful (G4)	3 [2, 4]	**< 0.001**	2 [1, 3]	**0.003**	2 [1, 3]
Enjoyed social life (G5)	3 [2, 4]	**< 0.001**	2 [1, 3]	0.628	2 [1, 2.25]
Needed extra help from family or friends (G6)	2 [1, 3]	**< 0.001**	1 [1, 2]	0.597	1 [1, 2]
**Work performance**					
Days in bed due to symptoms (W2)	2 [0, 5.5]	**< 0.001**	0 [0, 1]	0.480	0 [0, 1]
Decrease in routine activities (W3)	3 [0, 13.25]	**< 0.001**	0 [0, 3]	0.347	0 [0, 2]
Frequent rests at work (W5)	2 [1, 3]	**< 0.001**	2 [1, 2]	0.201	1 [1, 2]
Changes in usual job (W6)	2 [1, 3]	**< 0.001**	1 [1, 2]	0.327	1 [1, 2]
Decrease in hours of work (W7)	2 [1, 3.75]	**0.001**	1 [1, 2]	**0.011**	1 [1, 1.75]
**Sexual matters**					
Pain during sexual intercourse (S3)	2 [1, 2.25]	**0.035**	1 [1, 1]	1.000	1 [1, 1]
Satisfaction with sex life (S4)	2 [2, 3]	0.914	2 [2, 2]	0.564	2 [2, 2]
**Additional problems**					
Feeling of urinary tract infection (A1)	1 [1, 3]	**< 0.001**	1 [1, 2]	0.204	1 [1, 2]
Need for antibiotics (A2)	1 [1, 2]	**< 0.001**	1 [1, 1]	0.739	1 [1, 1]
Need for health professional help (A3)	1 [1, 2]	**0.014**	1 [1, 1]	0.132	1 [1, 1]
Need to visit the hospital (A4)	1 [1, 1]	**0.015**	1 [1, 1]	0.366	1 [1, 1]

^a^“*p*” values refer to symptoms at 2 weeks with DJ compared with baseline and were obtained using the Wilcoxon test

^b^“*p*” values refer to symptoms at 2 weeks with PSS compared with baseline and were obtained using the Wilcoxon test

^c^Question U11: “if you were to spend the rest of your life with the urinary symptoms, if any, associated with the stent just the way they are, how would you feel about it?” Answers: 5 “mostly dissatisfied”, 6 “unhappy”

^d^Pain scores rates refer only to the patients with pain

Statistical significant *p* values are reported in bold

## Discussion

This prospective longitudinal multicenter study focuses on SRS of patients with indwelling DJ undergoing substitution with a PSS after URS for stone treatment, showing a significant decrease of SRS after PSS placement, in terms of urinary symptoms, pain, general health and work performance. Despite routine stent insertion may not be necessary after uncomplicated URS [13, 14], several surveys have shown that ureteral stenting after URS remains commonplace in clinical practice [1, 15, 16]. Nevertheless, SRS still represent a major issue, with most of patients complaining of significant impact on quality of life [2, 3]. Double J distal end has been investigated as one of the potential factors influencing SRS, despite controversial results regarding the role of bladder material reduction [17, 18]. Vogt and colleagues developed a self-made PSS replacing the DJ distal loop with a 0.3 Fr suture reaching the bladder [6]. This device showed decreased SRS compared to conventional DJ in previous RCT [7–10], also providing unexpected dilation induced by the sutures in the distal ureter and orifice [6], less edema and histological inflammation in porcine and human models [19–21].

However, most of this evidence should be considered with caution, since the vast majority of previous studies involved self-made PSS [6–8, 10], with potential risks related to the modification of a medical device and poor reproducibility of results.

In their promising initial series, Vogt and colleagues involved 24 patients with indwelling DJ for heterogeneous reasons [6]—such as ureteral stones, UPJ obstruction and ureteral stenosis, strongly complaining of DJ-related symptoms, undergoing substitution with a self-made PSS, showing significantly decreased urinary symptoms and pain. Nevertheless, no subsequent studies investigated this scenario involving a commercially available PSS. Hence, this multicenter prospective longitudinal study was organized: to the best of our knowledge, this trial is the first on the topic involving a standardized device in DJ pre-stented unselected patients undergoing URS for stone treatment. Results showed significantly better scores in terms of urinary symptoms, pain and general health in favor of PSS, both considering USSQ scores and USSQ single answers. Moreover, the rate of patients reporting pain was lower for PSS, showing better work performance and quality of life.

The benefits in terms of SRS of this kind of PSS were previously shown in a RCT in non pre-stented patients [9]. The results provided by this longitudinal study demonstrate that this kind PSS can also reduce SRS in patients already bearing a DJ.

Since stent insertion after URS seems to be commonly used in urological practice [1, 15, 16, 22], Urologists may consider to use PSS after URS not only in naive patients, but also in pre-stented patients, at least in cases for which the Urologist decides against performing a stentless procedure. PSS may not only reduce SRS and improve quality of life compared to DJ, but also have a positive impact on social life and costs, being related to a better work performance compared to DJ.

Moreover, it has to be noticed that Pain Index Score, VAS Score and Sexual Matters Score were comparable to baseline with PSS, as well as many single items concerning pain (pain during physical activities and while passing urine, renal pain while passing urine, pain interfering with life), lifestyle (difficulty in light and heavy physical activities, enjoying social life, need for extra help from family members or friends, days in bed due to SRS, decrease in routine activities) and working habits (frequent rests at work, changes in usual work).

Further studies about PSS are desirable: a wider adoption of PSS in patients undergoing periodical stent substitution for UPJ obstruction or proximal ureteral stenosis [23, 24] may provide less SRS and more tolerability, while ensuring proper urine drainage. PSS group reported less renal pain during micturition and less impact of pain on everyday life, which might be particularly useful in chronic carriers of ureteral stent, potentially avoiding high renal pressure and consequent related adverse events [25, 26]. Nevertheless, due to the lack of long-term specific data, PSS should be cautiously employed in these settings, as well as in case of distal ureteral stenosis, regardless of previous evidence of dilation induced by the suture thread in the distal ureter [6].

Despite being the first study showing the benefits of DJ substitution with a marketed PSS in an unselected sample of pre-stented patients, this trial has several limitations. The first is related to its observational longitudinal nature. On the one hand, the accommodation of the ureter due to the indwelling DJ may have played a role in determining better PSS scores. On the other hand, the inflammatory and irritative response to DJ might have had a negative impact on PSS scores, thus limiting its effective benefits. To limit this potential bias, the promising results of the present study should be read with a view to conducting RCT comparing SRS in patients with DJ undergoing randomized substitution with DJ versus PSS after URS. In spite of a few drawbacks, this study provided additional significant data about PSS, which may induce Urologist to adopt this device in pre-stented patients after URS.

In conclusion, this study showed that patients undergoing DJ substitution with a PSS after URS report a significant decrease of SRS. Urologists may choose PSS after URS also in pre-stented patients to reduce the impact of SRS and to improve patients’ quality of life.

## Data Availability

The data that support the findings of this study are available on request from the corresponding author [AB].

## Abbreviations

DJ: Double J
PSS: Pigtail suture stent
RCT: Randomized controlled trials
UPJ: Ureteropelvic junction
URS: Ureteroscopy
USSQ: Ureteral Stent Symptom Questionnaire
